# Case report: Successful treatment with the combined therapy of interferon-alpha 2b and anlotinib in a patient with advanced hepatic epithelioid hemangioendothelioma

**DOI:** 10.3389/fmed.2022.1022017

**Published:** 2022-12-02

**Authors:** Xiaolei Liu, Ruiquan Zhou, Shuang Si, Liguo Liu, Shiwei Yang, Dongdong Han, Haidong Tan

**Affiliations:** Second Department of Hepatopancreatobiliary Surgery, China-Japan Friendship Hospital1, Beijing, China

**Keywords:** epithelioid hemangioendothelioma, liver, anlotinib, interferon, case report

## Abstract

Hepatic epithelioid hemangioendothelioma (HEH) is a very rare tumor originating from vascular endothelial cells, with unpredictable malignancy. At present, there is no standard treatment protocol yet established. Both surgical resection and liver transplantation have been reported to be effective treatments for HEH; however, multiple intrahepatic lesions or extrahepatic metastasis make these procedures unsuitable to most patients. Systematic therapy has also been investigated, but the results are undetermined due to the limited cases. Interferon-alpha 2b (IFN-a 2b) has also been used for the treatment of HEH. Based on our previous study, the rate of tumor regression with IFN-a 2b monotherapy was more than 50%. Here, we reported a patient with advanced HEH, who achieved a partial response with the combined therapy of anlotinib and IFN-a 2b. The tumor stayed stable for 2 years with anlotinib monotherapy and regressed 3 months after the combined therapy of anlotinib and IFN-a 2b. The synergistic effect of combined therapy with anlotinib and IFN-a 2b provided promising guidance for future clinical study.

## Introduction

Hepatic epithelioid hemangioendothelioma (HEH) is an extremely rare tumor that originates from vascular endothelial cells and has unpredictable malignancy (1, 2). Due to the rarity of the disease, clinical trials are very difficult to conduct. Currently, no standard treatment paradigm has been established yet. Surgical resection has been reported to be an effective treatment for HEH, but the risk of post-operative recurrence was very high, according to our previous study (3, 4). Moreover, most patients with HEH had multiple intrahepatic lesions or extrahepatic metastasis at the time of diagnosis, which limited the implementation of radical surgery (5). Liver transplantation (LT) has also been reported to be an effective treatment with favorable long-term outcomes (6). However, both the scarcity of organ donation and the high rate of extrahepatic metastasis limit the accessibility of LT for most patients with HEH. Meanwhile, the value of LT in HEH has also been doubted recently, considering both the risk of post-transplantation recurrence and the potential indolence of HEH (7).

Systematic therapy including chemotherapy, VEGF inhibitors, and immunotherapy have all been implemented in patients with HEH, but the results were undetermined due to the limited cases (8, 9). Interferon-alpha 2b (IFN-a 2b) has also been used for the treatment of epithelioid hemangioendothelioma (EH), which have the effect of innate and adaptive immune activation (10–13). According to our previous study of 42 patients with HEH with the treatment of IFN-a 2b monotherapy, tumor regression was achieved in more than 50% of patients, including two patients with complete response (CR), which was the most favorable results of systematic therapy ever reported (12). However, according to our experiences, the main defects of IFN-a 2b treatment were the slow reaction and the lack of efficacy for patients with HEH in critical condition. Anlotinib has been previously studied in patients with advanced sarcoma and the results showed satisfactory effectiveness with fewer side effects, ease of use, and acceptance by patients (14, 15). Considering the main defect of IFN-a 2b monotherapy and unpredictable malignancy of HEH, the combined therapy with anlotinib and IFN-a 2b may have the possibility of presenting synergistic effect for advanced HEH. Here, we report a patient with advanced HEH who achieved tumor regression after the combined therapy of anlotinib and IFN-a 2b. To the best of our knowledge, this is the first report of advanced HEH-achieved partial response (PR) with anlotinib and IFN-a 2b.

## Case presentation

**Figure 1:**
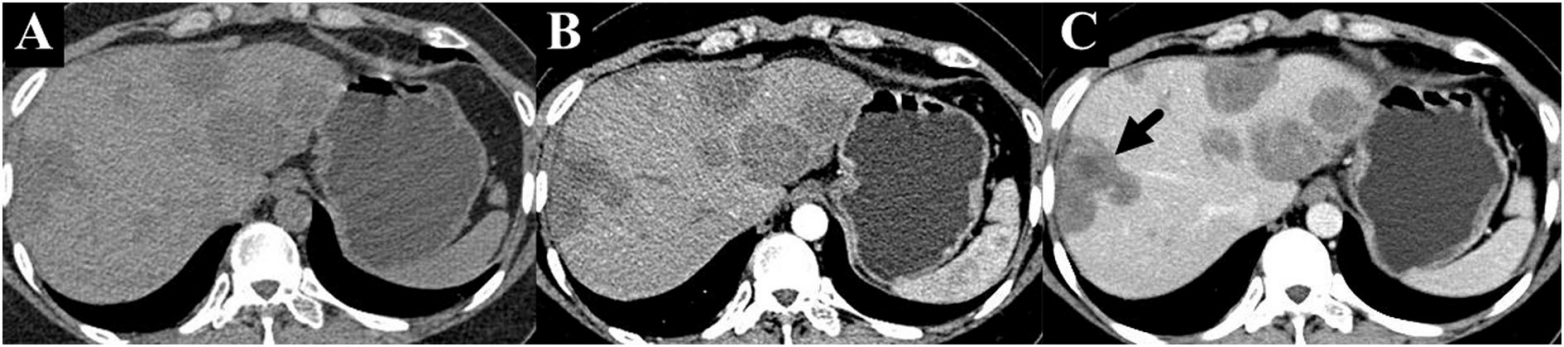
Unenhanced CT scan showed multiple intrahepatic lesions. **(A)** Contrast-enhanced CT scan showed heterogenous enhancement of the lesions with coalescence and target sign in the portal phase [**(B,C)**, marked with arrow].

**Figure 2:**
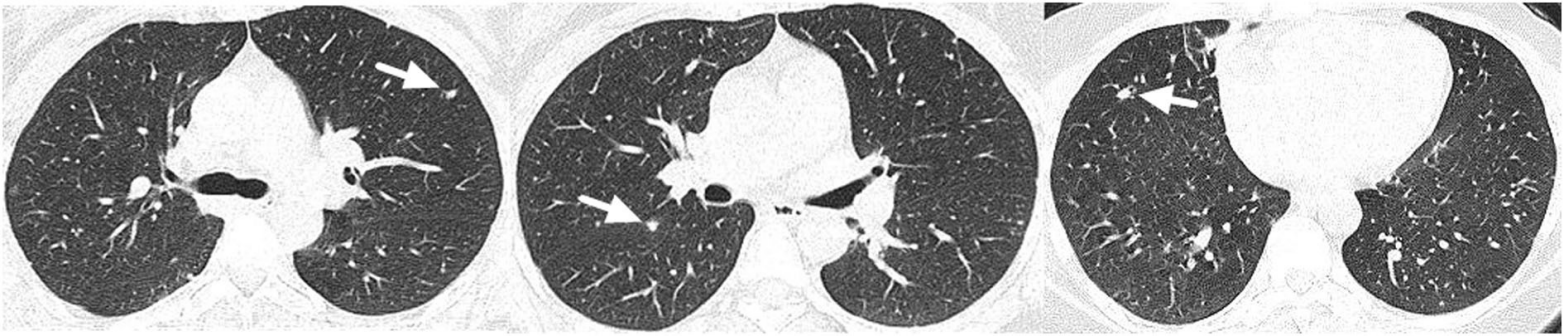
Multiple small lesions were detected in the lung (marked with arrows).

**Figure 3:**
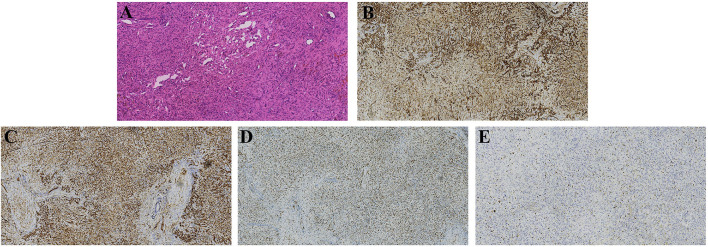
Hematoxylin and eosin and immunohistochemical staining of the liver biopsy, which showed spindle-shaped tumor cells and epithelioid tumor cells [**(A)**, hematoxylin and eosin, ×200], and positive for CD 31, CD34, and ERG [**(B–D)**, respectively, ×200]. The index rate of Ki-67 was 20% [**(E)**, × 200].

**Figure 4:**
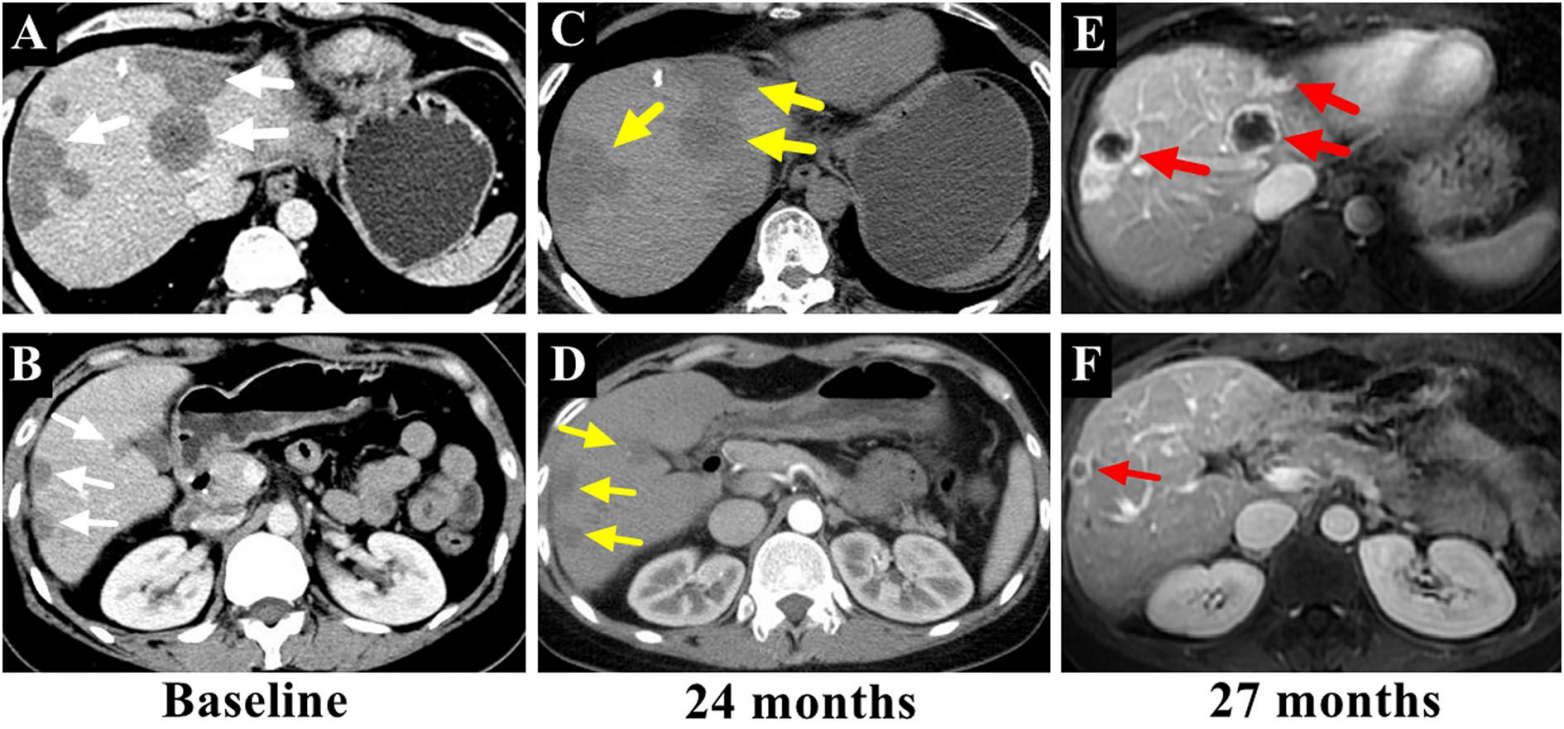
The intrahepatic lesions [**(A,B)**, marked with white arrows] were stable after 2 years of treatment with anlotinib monotherapy [**(C,D)**, marked with yellow arrows], while the lesions regressed or disappeared after 3 months of combined therapy with anlotinib and IFN-a 2b [**(E,F)**, marked with red arrows].

A 36-year-old female patient was referred to our clinic with occasionally detected multiple intrahepatic lesions by computed tomography (CT). The patient had no symptoms and no medical history of hepatitis or other diseases. No remarkable findings were revealed by physical examination. The initial blood tests showed normal blood cell counts and liver function. The serum levels of CA199, CEA, and AFP were also within the normal range. A contrast-enhanced CT scan showed multiple intrahepatic lesions with coalescence and target signs in the portal phase (Figure 1). A chest CT scan revealed multiple small lesions in the lung (Figure 2). According to the radiological appearances, HEH was considered and a liver biopsy was then conducted. Histological examination showed spindle-shaped tumor cells and epithelioid tumor cells (Figure 3). Immunohistochemical staining showed that the tumor cells were positive for CD31, CD34, and ERG, with a Ki-67 index rate of 20% (Figure 3). A histopathological examination confirmed the diagnosis of HEH. Surgical resection and LT were both excluded considering the multiple intrahepatic lesions and lung metastases. IFN-a 2b was suggested to the patient, and after consulting with the department of oncology, the patient chose the therapy of anlotinib. Anlotinib was then administered 12 mg once daily for 14 days with 7 days of rest thereafter. The patient was followed up every 3 months with lab tests of blood cell counts, liver, renal, and thyroid function, and the adverse event was recorded. The tumor response was assessed every 3–4 months by CT or magnetic resonance imaging (MRI). The patient had good tolerance of anlotinib monotherapy with no severe adverse event and the tumor was stable for 2 years. After revealing the long-term results of our study on IFN-a 2b in HEH, we discussed with the patient the possible synergistic effect of combined therapy with anlotinib and IFN-a 2b, and she accepted it. After 2 years of monotherapy of anlotinib, IFN-a 2b was then simultaneously administered by subcutaneous injection once every other day at the dose of 3 million units, as described in our previous study (12). Fever was reported after the first several shots of IFN-a 2b but gradually disappeared 10 days later. The patient also had slight fatigue and loss of appetite after the administration of IFN-a 2b. No other severe adverse event was reported. The intrahepatic lesions regressed obviously at 3 months after the combined therapy and the size of the largest lesion decreased from 4.8 to 3 cm (Figure 4). The metastatic lesions in the lung were stable. After 9 months of combined therapy, the size of the largest lesion was still 3 cm and the other intrahepatic lesions were all stable, compared with the images at 3 months of combined therapy. During the period of combined therapy, the patient had grade 1 anemia, leukopenia, and thrombocytopenia, but the therapy dosage was not adjusted. No abnormality was found in liver, renal, and thyroid function. Until now, the patient has been treated and followed up for 3 years with good physical status since the diagnosis of HEH.

## Discussion

HEH is an extremely rare intrahepatic tumor, usually detected occasionally by ultrasonography or CT with no clinical symptoms. Due to the differences in the biological behavior of the tumor, patients with HEH had a huge discrepancy in long-term survival (16). The radiological characteristics of HEH have been reported, such as a coalescent lesion, subcapsular lesion, capsular retraction, lollipop sign, and target sign (5, 17, 18). We have investigated the MRI appearances of 57 patients with HEH and the results showed that capsular retraction and lollipop sign were specific features of HEH, which could be used for differential diagnosis (5). Target sign has also been deemed as a radiological characteristic of HEH (19, 20). For this patient, the appearance of contrast-enhanced CT showed coalescent lesions and target signs in the portal phase, which indicated the possibility of HEH. Then, a histopathological examination by liver biopsy confirmed the diagnosis of HEH.

Currently, no standard therapy has been established for HEH yet. Although spontaneous tumor regression of HEH was reported, the results of our previous study showed that the tumor progressed gradually for most patients with HEH (3, 4, 21). LT has been reported to be an effective treatment for HEH with favorable long-term survival (6, 22). While considering the risk of post-transplantation recurrence and the potential indolence of HEH, the value of this procedure has also been doubted recently (7). Surgical resection has also been reported with good long-term results (1, 23). However, according to our previous study, surgical resection was impossible for most patients with HEH due to the multiple intrahepatic lesions (5). Therefore, the patients who achieved favorable long-term results after surgical resection only account for a small portion of the whole group. Moreover, our retrospective study showed that the risk of post-operative recurrence was also very high (4). Extrahepatic metastasis is very common in HEH, which also excludes the treatment choice of radical surgery and LT. Thus, for most patients with HEH, systematic therapy would be the only treatment option, including the patient we reported.

Due to the rarity of the disease, no large-scale clinical trial has ever been conducted for HEH. The clinical results of systematic therapies including chemotherapy, immunotherapy, and anti-angiogenesis targeted therapies, such as bevacizumab and oral tyrosine kinase inhibitors (sorafenib, lenvatinib, and pazopanib), have been reported based on case reports or small group of patients with HEH (24–26). Considering the biological discrepancy of HEH, the good therapeutic response of one patient cannot guarantee the same effect on others. Sirolimus has also been reported with a high rate of disease control both in adult and pediatric patients with EH (27, 28). However, the patients included in these studies had primary sites in the liver, lung, bone, and soft tissue, and the rate of tumor regression was just about 10% (27, 28). Anlotinib has been previously studied in patients with advanced sarcoma and the results showed satisfactory effectiveness with fewer side effects (14, 15). In this patient, anlotinib monotherapy achieved stable disease for 2 years with no severe adverse event, which provided another therapeutic option of target therapy for patients with HEH.

IFN-a 2b as immunotherapy has also been used to treat HEH. Although the mechanism was not clarified, innate and adaptive immune activation was speculated to be relative to the effect of IFN-a 2b treatment (13, 29). Our previous study investigated 42 patients with HEH with IFN-a 2b monotherapy and the results showed the rate of tumor regression was 52.4%, including two patients with CR (12). Moreover, the rate of 5-year survival was 97.2% with a median follow-up period of 33 months (12). Although the results could not guarantee the same effect on the whole HEH group, considering the discrepancy of biological behavior, the value of IFN-a 2b in the treatment of HEH should be noticed. After revealing the long-term results of IFN-a 2b, we suggested the combined therapy of anlotinib and IFN-a 2b to this patient, and the tumor regressed 3 months later. According to our previous study, the median time from the start of IFNa-2b monotherapy to tumor regression was 10 months. While for this patient, PR was achieved with 3 months of anlotinib and IFN-a 2b, which indicated the potential synergistic effect of IFN-a 2b and target therapy. The combined therapy was tolerated well by the patient and no adverse event was reported. The favorable outcome of combined therapy with anlotinib and IFN-a 2b further verified the value of IFN-a 2b in the treatment of HEH. IFN-a 2b has the potential to be used as a synergist for patients with HEH who achieve stable disease with target therapy. If the favorable results of long-term survival in HEH patients with IFN-a 2b monotherapy or IFN-a 2b combined with target therapy have further been confirmed by future studies, then the role and indication of LT in HEH should be reevaluated.

## Conclusion

In conclusion, IFN-a 2b has been studied to be effective in a large group of patients with HEH, while this is the first case report of a HEH patient with combined therapy of anlotinib and IFN-a 2b. The tumor was stable for 2 years with anlotinib monotherapy and regressed after 3 months of combined therapy of anlotinib and IFN-a 2b. The good response and safety of combined therapy with anlotinib and IFN-a 2b provide a promising guidance for future clinical study.

## Data availability statement

The original contributions presented in the study are included in the article/supplementary material, further inquiries can be directed to the corresponding author/s.

## Ethics statement

The studies involving human participants were reviewed and approved by the Ethical Committee of China-Japan Friendship Hospital. Written informed consent was obtained from the individual for the publication of any potentially identifiable images or data included in this article.

## Author contributions

XL, RZ, and SS acquired the data. LL, SY, and DH conducted the radiological analysis. XL primarily prepared the manuscript. HT revised the manuscript. All authors contributed to the article and approved the submitted version.

## Funding

This research was supported by the National High-Level Hospital Clinical Research Funding (Grant Number: 2022-NHLHCRF-PY-04).

## Conflict of interest

The authors declare that the research was conducted in the absence of any commercial or financial relationships that could be construed as a potential conflict of interest.

## Publisher's note

All claims expressed in this article are solely those of the authors and do not necessarily represent those of their affiliated organizations, or those of the publisher, the editors and the reviewers. Any product that may be evaluated in this article, or claim that may be made by its manufacturer, is not guaranteed or endorsed by the publisher.
